# Case Report: Severe Side Effects Following Treatment With First Generation Antipsychotics While Cariprazine Leads to Full Recovery

**DOI:** 10.3389/fpsyt.2021.804073

**Published:** 2021-12-14

**Authors:** Maris Taube

**Affiliations:** Department of Psychiatry and Narcology, Riga Stradinš University, Riga, Latvia

**Keywords:** cariprazine, full recovery, psychosis, neuroleptics, side effects, compliance, monotherapy

## Abstract

Schizophrenia is a psychiatric disorder characterized by positive, negative, cognitive and affective symptoms. Patient cooperation with health care professionals, compliance with the treatment regime, and regular use of medications are some of the preconditions that need to be met for a favorable disease course. A negative experience following the use of a first-generation antipsychotic to treat first-episode psychosis can negatively affect a patient's motivation for further medication use. In the clinical case reported here, cariprazine was able to restore one such patient's confidence in therapy and facilitated their cooperation with the physician, thereby ensuring effective control of negative and positive symptoms and good functioning for a period of 1 year. Cariprazine may be a good option for maintenance therapy following first-episode psychosis, especially in situations in which a patient has had a negative first experience associated with antipsychotic medication use.

## Introduction

Schizophrenia is a serious psychiatric disorder with a prevalence of ~1% in the population (1, 2). Positive symptoms of schizophrenia include delusions, hallucinations, abnormalities in how thoughts are linked together, thought insertion, withdrawal, thought broadcasting, and the belief that actions, feelings, or emotions are being controlled by external forces. Negative symptoms can include affective flattening, lack of motivation, loss of drive, lack of pleasure in any activities, poverty of speech, and diminished capacity to express feelings. Cognitive deficits may also be present with attention, language, and memory impairment. Affective symptoms may include depression and anxiety.

Throughout the course of schizophrenia, it is important to treat exacerbations of the positive, negative, and cognitive symptoms of the disease. Antipsychotics are effective in treating positive symptoms; however, treatment options for negative and cognitive symptoms are limited (3). Currently, most medications have not demonstrated sufficient efficacy in the treatment of negative and cognitive symptoms of schizophrenia; although, cariprazine shows promise for the treatment of negative symptoms (4–6).

Premature termination of therapy leading to repeated psychotic episode is a serious issue when treating schizophrenia. More than 50% of patients with schizophrenia terminate their treatment following hospital discharge (7). Each subsequent psychotic episode adversely affects the overall course of the disease and reduces the patient's functional abilities. However, patients with schizophrenia are less frequently rehospitalized if they receive maintenance therapy and outpatient treatment (8). Unfortunately, a patient's initial experience with a psychiatric service (e.g., admission to a psychiatric hospital, receiving first-generation antipsychotics, and experiencing the side effects of antipsychotics) may disincentivize them to continue with their treatment (9). While it is difficult to determine a patient's non-adherence to treatment early on; medication adjustment and the minimization of side effects could increase treatment adherence (10).

## Case Presentation

A 50-year-old male underwent emergency treatment for acute psychosis (delusions and hallucinations) in a psychiatric hospital and received haloperidol. The patient experienced the following side effects in the post hospital phase: acute dystonia, parkinsonism, dysarthria, and akathisia. The medication therapy was changed to a cariprazine-clozapine combination and was then continued with only cariprazine. A dose of 3 mg of cariprazine in monotherapy achieved stable improvement and full patient functionality for a period of at least 1 year.

### Background History

A family history uncovered mental health problems in a sister, which was likely depression. The patient was born in a difficult labor, and presented fetal macrosomia. At an early age, the patient experienced difficulty pronouncing words and had attended speech therapy. He had average grades in school and was a loner. He continued his education at the university and attained a doctoral degree. For the past 20 years he has worked at a public institution at a senior level position.

The patient divorced 15 years ago and has two children. He currently lives with his father and sister and has had a girlfriend for several years with whom he shares common interests in astrology and the occultism.

The patient had rarely been ill during his lifetime and indicated only a gastric ulcer as a problem. Approximately 5 years ago, he suffered a concussion, but did not incur permanent damage. He does not consume alcohol or other addictive substances.

### Diagnostic Assessment

An overview of events, medications, evaluation, and associated comments about first hospital treatment episode is found in Table 1. The patient was initially admitted to an acute psychiatric inpatient unit at the instigation of the family as he had rapidly—within a period of 1 week—developed acute psychosis, psychomotor agitation, and thoughts of being cursed.

**Table 1:** Timeline of patient events, medications and scoring (CGI-S, CGI-I) across the first inpatient treatment episode.

**Date**	**Event**	**Medication/s**	**CGI-S**	**CGI-I**	**Comment**
14/03/2020	Admission to psychiatric hospital observation unit	Diazepam (DZP) 10 milligrams daily (mg/d) intramuscularly (i/m)	6	4	Acute psychotic state; initial observation, blood, alcohol, COVID-19 test
16/03/2020	Transfer to acute psychiatric inpatient unit	Haloperidol (HAL) 7.5 mg/d i/m, trihexyphenidyl (THP) 6 mg/d orally (p/o), DZP 10 mg/d i/m	6	4	Acute psychotic state, working diagnosis: Paranoid schizophrenia according ICD 10 (delusion of influence). Differential diagnosis: Acute schizophrenia-like psychotic disorder (no enough information about duration of psychosis). Psychomotor agitation and acute exacerbation of psychosis were reasons for use of first generation antipsychotics
19/03/2020	Treatment in acute inpatient unit	DZP 5 mg/d p/o instead of i/m	5	3	Improvement, less psychomotor agitation
24/03/2020	Treatment in acute inpatient unit	Olanzapine (OLA) 5 mg/d p/o added, DZP canceled	5	3	Improvement, less psychomotor agitation
6/04/2020	Treatment in acute inpatient unit	HAL 15 mg/d p/o instead of i/m	4	3	Improvement, less psychomotor agitation
14/04/2020	Discharge from psychiatric inpatient unit	Recommendation to take HAL 15 mg/d p/o, THP 6 mg/d p/o and OLA 5 mg/d p/o	3	2	Final (discharge) diagnosis: schizophrenia according ICD 10 (delusion of influence, time criteria: more than 1 month)

Using the International Statistical Classification of Diseases and Related Health Problems 10th Revision (ICD 10) (11), a diagnosis of paranoid schizophrenia (F20.0) was made. Organic causes such as drug-induced psychosis, delirium, and metabolic disturbances were excluded. Differentiation from acute schizophrenia-like psychotic disorder (F23.2) was made based on detailed information from the patient and relatives concerning the duration of the psychosis. At the inpatient unit, the patient received haloperidol up to 15 mg/day (which was initially given intramuscularly in a dose 7.5 mg/day and then perorally 15 mg/day), 6 mg/day of trihexyphenidyl, and 5 mg of olanzapine in the evenings. The patient remained hospitalized for 31 days. Throughout this period, his acute psychotic symptoms lessened, although they were not eliminated. Some delusions remained, and the patient was suspicious.

The patient underwent a psychodiagnostic examination, and it was noted that his thinking was distinctly peculiar, atypical, and characterized by making judgments on the basis of assumptions understandable to himself but difficult for others to understand. The personality profile reflected fatigue, an apathetic state, a low energy level, and difficulty in motivating himself with purposeful actions. Interpersonal relations showed a tendency toward social introversion with avoidance and distancing behavior, a limited ability to express feelings and experiences, sensitivity to other people's attitudes toward him, cautiousness, and slight suspiciousness.

The patient was discharged from the hospital with recommendations to take 15 mg/day of haloperidol, 6 mg/day of trihexyphenidyl, and 5 mg of olanzapine in the evenings. The patient had planned to return to work. However, his condition deteriorated within ~2 weeks following hospital discharge. An overview of events, medications, evaluations, and comments about second hospitalization and outpatient treatment is available in Table 2. The patient began to exhibit side effects from the neuroleptics including parkinsonism, akathisia, dysarthria, and acute dystonia. This was partially the result of the patient reducing the dosage of trihexyphenidyl. In addition, without full understanding about the role of medications, he had little fluid intake due to a fear of sweating. He sought help from the outpatient service because of the pronounced neuroleptic side effects. His treatment was subsequently adjusted, and he was rehospitalized.

**Table 2:** Timeline of patient events, medications and scoring (CGI-S, CGI-I) across the outpatient treatment and second inpatient treatment episode.

**Date**	**Event**	**Medication/s**	**CGI-S**	**CGI-I**	**Comment**
30/04/2020	Outpatient visit	HAL 15 mg/d p/o, OLA 5 mg/d p/o and THP 2 mg/d p/o	3	4	The patient reduced the dosage of THP, reduced fluid intake Extrapyramidal side effects: parkinsonism, akathisia, dysarthria and acute dystonia are presented
5/05/2020	Admission in psychiatric nonacute inpatient unit	Cariprazine (CAR) 3 mg/d p/o, clozapine (CLO) 6.25 mg/d p/o, THP 6 mg/d p/o, DZP 10 mg/d intravenously (i/v) and DZP 5 mg/d p/o	3	4	CLO was added to avoid psychosis as result of rapid changing of medicines from first generation antipsychotics to CAR
8/05/2020	Treatment in psychiatric non-acute inpatient unit	CAR 4.5 mg/d p/o, CLO 6.25 mg/d p/o, THP 3 mg/d p/o, DZP 10 mg/d i/v and DZP 5 mg/d p/o	3	4	Psychological counseling, drama, music and visual art therapy, ergotherapy were added to the treatment plane, less extrapyramidal side effects, downsizing of THP
12/05/2020	Treatment in psychiatric non-acute inpatient unit	CAR 4.5 mg/d p/o, CLO 6.25 mg/d p/o, THP canceled, DZP 10 mg/d i/v and DZP p/o canceled	3	3	Improvement, reduced extrapyramidal side effects
15/05/2020	Treatment in psychiatric nonacute inpatient unit	CAR 6 mg/d p/o, CLO 12.5 mg/d p/o, DZP i/v canceled	3	3	Improvement, reduced extrapyramidal side effects
18/05/2020	Treatment in psychiatric nonacute inpatient unit	CAR 6 mg/d p/o, CLO 25 mg/d p/o	3	3	Improvement, reduced extrapyramidal side effects
20/05/2020	Treatment in psychiatric nonacute inpatient unit	CAR 6 mg/d p/o, CLO 37.5 mg/d p/o, THP 2 mg/d p/o	3	4	The patient complained about tremor
29/05/2020	Discharge from psychiatric nonacute inpatient unit	CAR 6 mg/d p/o, CLO 37.5 mg/d p/o	2	2	Loss of drive and diminished capacity to express feelings are mildly pronounced
30/06/2020	Outpatient visit	CAR 6 mg/d p/o, CLO 25 mg/d p/o, THP 2 mg/d p/o	2	2	
12/08/2020	Outpatient visit	CAR 6 mg/d p/o, CLO 25 mg/d p/o, THP canceled	2	2	No tremor or other extrapyramidal side effects
13/10/2020	Outpatient visit	CAR 6 mg/d p/o, CLO 25 mg/d p/o	2	2	
15/12/2020	Outpatient visit	CAR 6 mg/d p/o, CLO canceled	2	2	No risk for psychotic episodes, CLO canceled
16/02/2021	Outpatient visit	CAR 6 mg/d p/o	2	2	
8/04/2021	Outpatient visit	CAR 4.5 mg/d p/o	2	2	
3/06/2021	Outpatient visit	CAR 3 mg/d p/o	2	2	
6/08/2021	Outpatient visit	CAR 3 mg/d p/o	2	2	
15/10/2021	Outpatient visit	CAR 3 mg/d p/o	2	2	Partly diminished capacity to express feelings and reduced ability to persist in goal-directed behavioral (e.g., rent a new flat) still exist. Possibility to cancel medicines was discussed with the patient

Hospital treatment consisted of intravenously administered diazepam to alleviate the side effects, stopping the administration of haloperidol and olanzapine and, instead, introducing 6 mg/day of cariprazine with 37.5 mg of clozapine in the evenings, and 6 mg/day of trihexyphenidyl. Clozapine was added to avoid psychosis as result of the rapid changing of medicines from first generation antipsychotics to cariprazine. Given a treatment history that included an acute psychotic episode, possible future monotherapy appeared to be unlikely. A more likely option was a combination of cariprazine and clozapine. However, the patient received complex therapy during his hospital stay, including psychological counseling, drama, music, and visual art therapy (12), as well as ergotherapy sessions. The medication side effects resolved, and the patient regained confidence in therapy. Therefore, 6 mg/day of cariprazine was recommended following hospital discharge.

### Current Status

After discharge, the patient returned to work. He currently sees a psychiatrist on a regular basis, and the dose of cariprazine has been gradually reduced to 3 mg/day in monotherapy. To date, his condition is stable. He has been fully functional for a year, with no positive or negative symptoms such as a loss of drive. However, a diminished capacity to express feelings are mildly pronounced. No additional psychological and social therapies have been needed. The patient is positive about his future treatment course; although, no final decision has been made concerning future medication use. While the patient is interested in quitting medication, there is the risk of future psychotic episodes or, in the case of a worsening mental health status, he might avoid treatment based on his negative experience.

## Discussion

After analyzing the patient's disease course, there is evidence that, while the psychotic episode developed rapidly, there were premorbid signs suggestive of negative symptoms. His acute psychosis and agitation did not allow for a treatment method other than the use of first-generation neuroleptics (13). However, during the treatment process, the administration of olanzapine (14) was initiated with the aim of further transitioning to the use of second-generation antipsychotics.

Cooperation with a psychiatric patient is crucial for a successful treatment outcome. A patient's confidence in therapy is also important, and medication side effects do not facilitate trust in treatments regiments. However, negative, initial hospital treatment experiences can be avoided if proper information and psychoeducation is provided to the patient. It is possible that other atypical, antipsychotic medications would yield results that are similar to cariprazine in regard to negative side effects. However, the choice of cariprazine in this case was based on the patient's negative symptoms.

The patient was at high risk for avoiding further treatment given his negative experience following his first-episode psychosis. Therefore, further treatment needed to result in significantly fewer (and less severe) side effects, but with sufficient efficacy in addressing both his positive and negative symptoms. One important therapy goal is preventing recurrent psychotic episodes that can lead to deterioration in the overall course of the disease. In this case, the patient had a number of preconditions which suggested a sufficiently favorable disease course was possible. This included a late onset of psychosis, a high level of educational attainment, employment, and acute psychosis development (15). It was also extremely important for the patient to have confidence in further treatment and to trust the health care professionals. The choice of cariprazine proved to be effective as the patient's mental health and social functioning have been adequate for a year and continues to be stable.

There are several limitations concerning the approach taken toward this case. First no other clinical scales were used for patient assessment other than the Clinical Global Impressions Scale-Severity (CGI-S) and the Clinical Global Impressions Scale- Improvement (CGI-I). Second, during the first inpatient treatment course not enough information was provided to the patient. There was a low level of psychoeducation and not enough information was given regarding the role of each medication and his future treatment. Providing this information must play role for successful treatment. Finally, the patient did not receive any antipsychotic medications other than cariprazine, haloperidol and, periodically, olanzapine and clozapine. Therefore, we cannot know the possible results of medications other than cariprazine.

Cariprazine should be considered an antipsychotic of choice for maintenance therapy in patients who have experienced first-episode psychosis, especially if the patient has had a negative experience associated with medication side effects.

## Patient Perspective

The patient is fully satisfied about current treatment, but also remember side effects from first generation antipsychotics. He is still cautious about treatment and hope quitting medication in a near future. The patient is positive about future, employment, relationship.

## Data Availability Statement

The original contributions presented in the study are included in the article/supplementary material, further inquiries can be directed to the corresponding author.

## Ethics Statement

The patient provided written informed consent. Institutional approval was not necessary for publication.

## Author Contributions

MT designed the case report, gathered the data, wrote, and edited the manuscript.

## Funding

The open access fee is covered by Prescott Medical Communications Group.

## Conflict of Interest

The author has received financial benefits for participation in boards, and as a speaker from the pharmaceutical companies: Lundbeck, Janssen-Cilag, Gedeon Richter, Olainfarm, Grindex, and Medochemie.

## Publisher's Note

All claims expressed in this article are solely those of the authors and do not necessarily represent those of their affiliated organizations, or those of the publisher, the editors and the reviewers. Any product that may be evaluated in this article, or claim that may be made by its manufacturer, is not guaranteed or endorsed by the publisher.
